# Who We Test For: Aligning Relational and Public Health Responsibilities in COVID-19 Testing in Scotland

**DOI:** 10.1080/01459740.2024.2349514

**Published:** 2024-05-07

**Authors:** Imogen Bevan, Linda Bauld, Alice Street

**Affiliations:** a School of Social and Political Science, University of Edinburgh, Edinburgh, UK; b Usher Institute, University of Edinburgh, Edinburgh, UK

**Keywords:** COVID-19, diagnosis, pandemic, public health, Scotland, testing

## Abstract

COVID-19 testing programs in the UK often called on people to test to “protect others.” In this article we explore motivations to test and the relationships to “others” involved in an asymptomatic testing program at a Scottish university. We show that participants engaged with testing as a relational technology, through which they navigated multiple overlapping responsibilities to kin, colleagues, flatmates, strangers, and to more diffuse publics. We argue that the success of testing as a technique of governance depends not only on the production of disciplined selves, but also on the program’s capacity to align interpersonal and public scales of responsibility.

I wasn’t concerned about other people necessarily passing it [COVID-19^1^] on to me, I was thinking, “If I’m going in [to work], I don’t want to be the one that spreads it around everyone by unknowingly being positive” […]. So it was just that reassurance, knowing that at least twice a week you were being tested, and then if you were seeing people outside of the university environment you could be like, “Well, I’ve been tested at work this week, we can see each other and I feel like I’m not going to be a risk to you.” (Matthew, postdoctoral researcher in biological sciences, November 2021)

In November 2021, deep into the second year of the COVID-19 pandemic in Scotland, Matthew, a postdoctoral researcher, was explaining why he had enrolled in the University of Edinburgh’s pilot asymptomatic testing program for COVID-19, TestEd. As for other participants we interviewed, Matthew described participation in the testing program as a way to protect friends and colleagues, and to manage responsibility for the risk his potentially infectious body posed to them. Harris, a professor in natural sciences, similarly told us, “I just feel that like with lots of areas of life, you’ve got to act responsibly. It comes with driving carefully and trying not to upset people and lots of things you do in life, unless you’re a sociopath, you’re conscious of your effect on other people.” Both interviewees situated testing on a continuum of interpersonal responsibilities. Yet Matthew stressed that he tested not only to protect friends and colleagues, but “because you don’t know whether *they* are then going to see other relatives,” (his emphasis).^2^ In considering how his actions would affect people’s future interactions, this mode of thinking reconfigured social relationships as chains of disease transmission, revealing the extent to which people’s ways of relating had become inflected by what others have termed an “epidemiological consciousness” (Long et al. 2023:21) or kinds of “epidemiological reasoning” (Engelmann 2021:104). Equally importantly, Matthew was referring to the problem that his social, yet potentially viral, body posed to his friends’ capacity to protect their relatives – pointing to the primacy of duties toward kin in a pandemic. In a context of sustained scientific, political and social uncertainty, public health tools designed for case-finding and controlling disease prevalence at the level of particular populations interacted with people’s uses of testing as a solution to the interpersonal problems and relational dangers caused by pandemic living.

By the time TestEd was introduced, in January 2021, COVID-19 testing was well-established as a technique of pandemic governance across the UK nations and had played a key role in people’s experiences of pandemic living with others. Across the course of the pandemic, testing had served a multitude of purposes, shaped by a combination of what was technically feasible, available resources, political pressures, and changing clinical and public health needs. These ranged from tracing the source of an outbreak, to aiding clinical management of individual patients, generating surveillance data, and supporting public health interventions such as the isolation of positive cases and their contacts. In Scotland, testing technologies and rationales multiplied and changed in the first two years of the pandemic. Early attempts to deploy testing in hospitals to aid clinical management but also identify outbreaks and contain the spread of the virus were hampered by a lack of laboratories and resources. When Scotland’s first lockdown was announced in March 2020, the testing strategy had shifted from “containment” to “delay” (Scottish Government 2020). Test and Protect, Scotland’s hastily configured testing and contract tracing system,^3^ offered free PCR testing to anyone with symptoms of COVID-19, and positive cases and their contacts were instructed to self-isolate for a specified, but frequently changing, number of days, with the dual aim of minimizing community transmission and generating local and national prevalence data. Thousands of people also participated in a government surveillance survey to track prevalence in the community. By autumn 2020, as vaccines were being rolled out, the political response shifted from a focus on minimizing transmission through restrictions on public movement and interaction, to the need to safely reopen the economy, including educational institutions. Lateral flow testing devices (LFDs), which were cheaper but less accurate than PCR testing, provided rapid results in 15 minutes, and could be used at home, now took center stage. In Scotland, LFDs were made freely available to target groups from September 2020, then to the general population from April 2021. Members of the public were asked to test twice a week, with a focus on the negative result as a free pass to return to work and socialize in public spaces.

Launched at the University of Edinburgh in a limited number of campus locations in January 2021, and rolled out across the University in April 2021, TestEd was intended to contribute to supporting the reopening of the economy through the provision of an affordable and convenient asymptomatic testing service for staff and students. For university managers, the TestEd project was intended to facilitate a return to in-person teaching and establish a “new normal” within a fully functioning institution, while as a research project it aimed to provide a potential blueprint for workplace and population-based screening programs in the future. Proposing to operate both through measuring prevalence and case finding, signposting positive persons toward NHS testing routes, TestEd offered to govern both at the level of the university population and the individual.

Many of the public health interventions introduced in the UK during the COVID-19 pandemic, such as face masks or vaccines, worked bi-directionally, and protected the health of the person who adopted them in addition to that of those around them. By contrast, asymptomatic testing stood out in its exclusive focus on the protection of others, since a person who tested positive did not receive medical care or protection (unless they became unwell) but was instead required to confine themselves to their home and curtail their in-person interactions.^4^ As a tool that exclusively targeted infection at the level of the population – yet whose functionality depended on the voluntary actions of symptom-free individuals – asymptomatic COVID-19 testing differed from most population screening programs that members of the UK public were familiar with before 2020, such as cancer screening, where a positive result can facilitate early preventative action and/or access to treatment. Those screening programs neatly align the “anatomopolitics” of biomedical care for the individual body with the “biopolitics” of governing life in the “body” of the population (Foucault 2003). By contrast, voluntary asymptomatic COVID-19 testing programs, where the ultimate purpose was to bring down numbers at a population level rather than deliver individualized care, depended on the willingness of individuals to accept the “hidden burden” of testing (Street et al. 2022:1) for reasons other than personal health.

In this article we draw on 48 in-depth interviews with University staff and student volunteers, who enrolled in the TestEd program in Edinburgh between January 2021 and February 2022, to explore the dual role of asymptomatic testing as both a highly individualized self-testing technology and a public health intervention. A substantial field of research in the anthropology and history of epidemics has explored the relationships of governance and citizenship inscribed in techniques of epidemic control (Arnold 1993; Biehl 2007, Chowdhury and Basu 2021; Lakoff 2017; Lynteris 2018). This work has shown that epidemic governance frequently operates through techniques of individualization and responsibilisation. In the UK, COVID-19 tests were just one among a range of voluntary behavioral interventions that impressed on people their civic duty to contribute to infection control (Andreouli and Brice 2021; Herrick 2022). The UK government’s COVID-19 public health messaging, also deployed in Scotland, operated along the lines of personal responsibility, “fear appeals” and “moralising messages” (McClaughlin et al. 2023:2), that placed blame for viral prevalence and pressures on national health services on individuals – an approach critiqued as having “deflected attention from the broader systemic failures that characterized the UK’s pandemic response” (Cooper et al. 2023:11). The National Health Service (NHS)’s “Stay Home, Protect the NHS, Save Lives” and “Can you look them in the eyes?” television and poster campaigns launched in 2020 and 2021, urged people in Scotland and England to comply with lockdown measures and protective behaviors: hand washing, social distancing, mask wearing, testing when symptomatic, and self-isolating. Through these behaviors, individuals were encouraged to take responsibility, in order to “Protect the NHS,” “Protect your community,” or simply “For your family,” “For your friends.”^5^ Messaging around vaccines likewise emphasized the moral duty to avoid spreading the virus to family and “those around you” (Public Health Scotland 2022:5). More ambiguous deployments of scale included, “Keep everyone safe,” “People will die,” “Anyone can spread it.”^6^ Linking scales of protection and responsibility between the interpersonal, community and the level of the state (family/community/NHS) appeared to form an important aspect of public health communication strategies.

Rather than focus on the political rationales and public health logics that underpin techniques of governance and responsibilisation, in this article, we seek to build on a growing body of work that explores the motivations, intentions and experiences of people who are targeted by such interventions (e.g. Fearnley and Wu 2023; Hanna et al. 2022). Where much of this research has focused on resistance (e.g. Fairhead 2016), we explore the social and ethical orientations of people who voluntarily participated in – rather than opposed – a program of epidemic control. We ask how people navigate the different kinds and scales of relationship that are constituted through asymptomatic testing, and the “ordinary” or “everyday” ethics (Das 2007; Lambek 2010; Mattingly 2014) involved in testing for others. We are interested in both the extent to which those testing relations reflect a “domestication” of public health logics and epidemiological reasoning (Engelmann and Montgomery 2020), and the insight they offer to altered norms of intimacy, forms of sociality, and modes of care during the COVID-19 pandemic (Dawson and Dennis 2020; Herrick 2022; Levine and Manderson 2021; Twamley et al. 2023; Watson et al. 2021).

In doing so, we attend to testing as a “relational technology” (Street 2012:1, 2014) that both reveals and transforms relationships between persons, and to testing as “relational action” (Stasch 2009:17), where the nature of social relationships and relational categories are established through events. Where these concepts have usually been employed to understand interpersonal relationships, for example between patients and doctors in a hospital or between kin, we suggest that in the UK’s COVID-19 pandemic, testing operates not only to “test roles, relationships, and responsibilities” (Robinson 2020:460) between persons, but also to align relationships at different scales, in this case interpersonal relationships, relationships to strangers and relationships to more diffuse “publics.” We describe spaces where testing for the health of social relationships and for the health of the population come into alignment within the simple act of undertaking a test. But crucially, we highlight the unstable nature of alignments between different levels, scales and temporalities – and the work required to achieve overlap (Casper and Clarke 1998; Clarke and Fujimura 1992; Engel 2020; Fujimura 1987; Cross and Street 2022). More fundamentally, we argue that the capacity for alignment across relational scales is essential to the utility of asymptomatic testing as a public health tool.

We begin by exploring participants’ engagement with TestEd as a relational technology through which they mediated the constantly changing obligations and responsibilities that specific relationships imposed on them in the context of a pandemic. We go on to show that participants encountered TestEd as part of a constantly shifting assemblage of testing possibilities or “ecosystem” (Yellapa et al. 2017:1). Participants often configured for themselves an ad hoc, highly personalized, diagnostic system, with which to meet multiple relational demands. These personal testing systems blurred the distinctions between asymptomatic/symptomatic or diagnostic/screening tests, exposing tensions between the different purposes and scales of testing, and in some cases, putting the goals of public health interventions at risk. We demonstrate that what and who testing technologies are for (Lasco 2022) – in public health terms their “use case” – is open to reinterpretation and re-design in relation to their user’s everyday social engagements and practices. This has important implications for testing technologies’ capacity to align the goals of epidemic governance with personal understandings of responsibility.

## Methods

The TestEd program^7^ ran for two years, from January 11, 2021 to December 18, 2022, with a total of 9,201 of the University of Edinburgh’s students and staff enrolled and 168,174 samples tested. We draw on 48 interviews with TestEd participants, carried out between May 2021 and February 2022 as part of the wider TestEd project, a collaboration between biomedical, physical, clinical and social scientists along with health economists. The social science team, made up of behavioral scientists, epidemiologists, sociologists and anthropologists, had the primary objective of conducting surveys and interviews to examine the public health aspects of COVID-19 testing, including facilitators and barriers to testing, and adherence with public health guidelines, in particular those surrounding self-isolation (Bauld et al. 2023). Interviews took place remotely via Microsoft Teams, Zoom, or phone call due to COVID-19 restrictions. Interviews covered a range of topics, including people’s wider experiences of the pandemic, their testing stories and the place of testing in their everyday lives.^8^

Participants who tested positive within TestEd were asked for consent to be contacted for a follow-up interview. Between May and December 2021, we prioritized interviews with participants who tested positive, resulting in 33 interviews. These were supplemented by 15 interviews between December 2021 and February 2022, with participants who had consistently tested negative and had participated in a TestEd survey, during which they had given consent to be contacted for a follow-up interview. We used purposive sampling to recruit participants for this second phase among a wider range of demographic groups, with attention to university role, age, gender, ethnicity and disability.^9^ Author 1 also enrolled as a student participant and took part in TestEd; this article is also informed by her experience.

## The pandemic university

No need for a swab up the nose or at the back of the throat, you just need to give a saliva sample. It’s quick, easy and accurate, but it needs students like you to make it work. Get involved. (Newsletter to students from University management, 16 November 2021)

As this quote from TestEd’s promotional materials indicate, the technology promised several main advantages in relation to existing public health tools. The technology offered a saliva-based sample collection method that was more comfortable to the user than the nasal/nasopharyngeal swabbing method that characterized the free lateral flow device tests (LFDs) provided by the UK government for asymptomatic testing. TestEd’s use of PCR technology made these tests more accurate than LFDs, which tended to have low sensitivity (ability to pick up positive cases). PCR was usually costly, whereas the pooled method^10^ meant TestEd could offer highly accurate and convenient testing at a comparable cost.

TestEd’s potential value as a public health tool also derived from its situatedness within a large public university. Throughout the COVID-19 pandemic in the UK, higher education emerged as a particularly problematic space of public health governance – and a public good requiring protection. The social density of spaces including university accommodation, lecture halls and social meeting spaces, and the perceived social promiscuity of the student sub-population were viewed as opportunities for campus-based outbreaks (e.g. Brooks-Pollock et al. 2021; Hill et al. 2021). The “leakiness” of the institution via staff households, student employment and socializing, and student migration at the beginning and end of semesters was perceived as posing a risk of “spillover” to other populations, potentially contributing to an overall increase in national level prevalence (Aggarwal et al. 2022; Enright et al. 2021). Universities frequently featured in the national media, in government announcements and in SAGE meeting minutes (e.g. SAGE 2020) as an especially challenging space in which to control infection and potentially disease.

**Figure 1.**
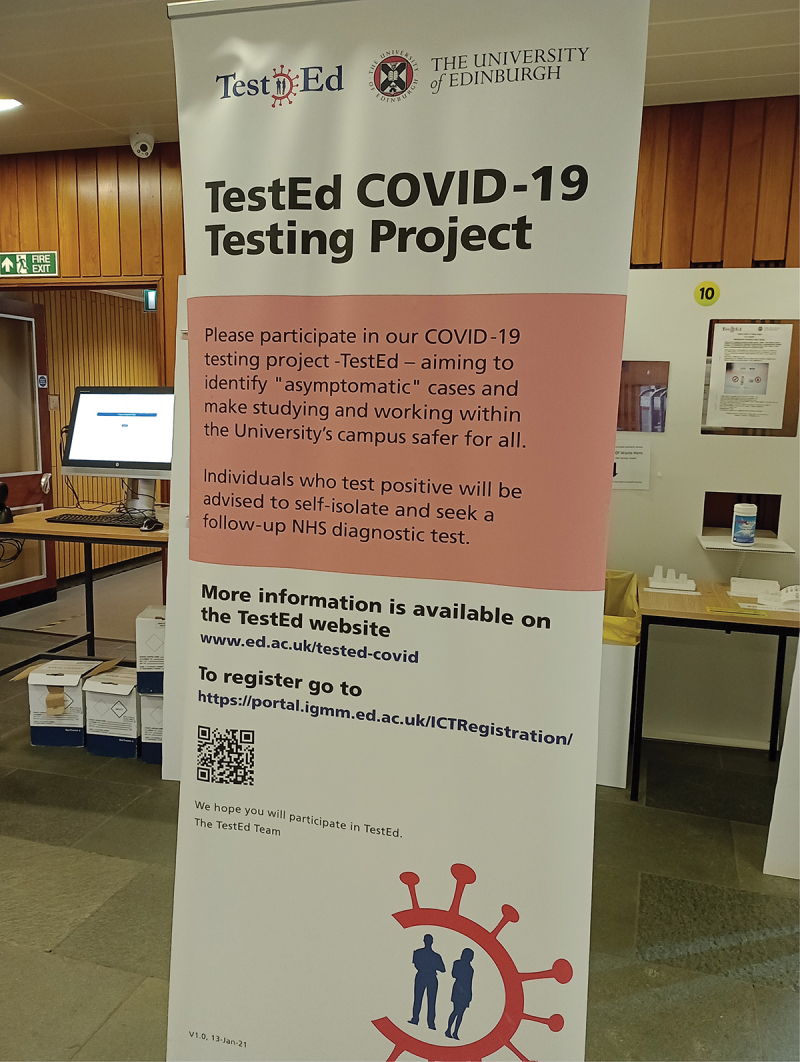
TestEd station, Main Library (photo by Imogen Bevan, 2022).

When the first national lockdown due to COVID-19 was announced in Scotland and other UK nations in March 2020, University campuses were closed except for essential research, teaching and maintenance. Campuses were partially re-opened in September 2020, accompanied by an assemblage of public health guidance and hybrid teaching arrangements – including mask wearing, hand sanitizing and ventilation protocols, limits on numbers of persons in enclosed spaces, and one-way systems, to ensure the safe functioning of the university. In this context, TestEd was envisioned as a desirable public health tool. “[I]t needs students like you to make it work,” the promotional materials suggested – where “working” referred to the project’s ability to bring down numbers of cases at the level of the University population, and potentially the wider community, as well as to the optimization of the testing method and associated user platform. In January 2021, the first TestEd sample collection booths were funded and set up by the university, and in April 2021, a COVID-19 Medical Research Council grant^11^ for innovative responses to COVID-19 allowed the group to proceed with formally piloting TestEd and to expand to 31 sample collection sites across the university, offering the program to all those who were regularly working or studying on campus (Figure 1).

For TestEd participants, the testing program was introduced amid an unprecedented experience of political and personal upheaval. Staff and students had lived through the early months of the pandemic, when Scotland had faced high rates of morality and hospitalizations, followed by almost a year of restrictions, including multiple periods of national and more localized lockdowns. The interview period, almost a year since the start of the pandemic, was characterized by an ongoing atmosphere of uncertainty – about how to relate, about how long such ways of living might continue. In the second half of 2021, most of the adult population had received one dose of a vaccine to protect them from the most severe outcomes of a COVID-19 infection and a collective return to work was underway.^12^ Participants were making sense of the constantly evolving patchwork of epidemic technologies in place: face coverings continued to be mandatory in public indoor settings at the time of interviews, while restrictions on physical distancing and limits on numbers of people meeting indoors had ended in summer 2021.^13^ This regulatory landscape continued to shift, with the introduction of vaccine passports, and new spikes and falls in case numbers. Participants interviewed in December 2021 and January 2022 saw an intensification of measures, with the return of strict restrictions on travel, physical distancing, and reduced numbers of households meeting indoors.

In this context of ever-changing tiers, regulations and guidelines, and with the potential infectiousness of one’s body an ever-present possibility, navigating the ethics and etiquette of everyday relationships and interactions had become extremely complex. With the return of the student body to campus, and government discourses about a needed return to “normality,”^14^ many staff and students we spoke to expressed their desires for such a public health tool. None, however, suggested that the University ought to remain closed, reflecting the balance at that time between population health, education and the national economy as different kinds of “goods.” In participating in TestEd, they adopted public health framings of the university as a risky space, and to some degree, ambivalence about students as a risky population within the context of this necessary return. For example, lecturer and clinical service provider, Elana, recalled how in the summer months of 2020 she had felt “very safe” in the hospital, when her team had been divided into bubbles, and were following strict disinfection protocols. But after a summer characterized by restrictions including a maximum of three households meeting indoors, the mass return of students to the hospital for teaching purposes had reduced this sense of safety. While she trusted her colleagues from the service to have “very similar lifestyles” and styles of care as herself, the students were “young, and they may not understand the consequences,” she reflected.

The [rare] times I went to Edinburgh, I could see young students meeting up. And it’s like, “Ah you are not allowed to meet, you could be my students.” So yes, again it may be totally unfair and it may be generalizing and that maybe the students were actually really good, it’s just I cannot be sure.

Knowledge that the SARS-CoV-2 virus was transmitted between bodies via contact with surfaces, droplets and aerosols had transformed people’s relationships to university spaces, which had become refracted through evaluations of the infection risk that people posed to one another (Trnka et al. 2021). Perceptions of students as problematically social – which reflected representations of students in the national media – exemplify how public health logics and diagrams had social lives (e.g. Lynteris 2017; Rhodes and Lancaster 2020), could be absorbed or “domesticated” (Engelmann and Montgomery 2020) and integrated into experimental forms of “epidemiological sociality” (Long et al. 2023:22). While many TestEd users were concerned about navigating the University as a high-risk space in terms of potential “close contacts,” their use of testing was incorporated into everyday strategies for pandemic living in more multiple ways that went beyond these epidemiological logics, as we go on to show.

## Testing for ‘publics’ and ‘communities’

Participants gave multiple reasons for participating in TestEd. Some directly identified with the public health goals of the program, describing participation as a way to limit their personal contribution to outbreaks on campus and beyond. Carla, a postdoctoral researcher working in the laboratory, spoke of avoiding “some kind of big spreading event” at work, when we spoke to her in July 2021, a time when lower case numbers meant fewer restrictions in place. Hamish, a first-year undergraduate in social sciences felt equally concerned in December 2021. With case numbers spiking due to the spread of a new variant with yet unknown health implications, strict restrictions on travel, physical distancing, and numbers of households meeting indoors had been re-introduced. Hamish told us, “I’m not afraid for my own wellbeing or long-term health, but I am concerned that I suppose I don’t want to contribute to spreading the pandemic any further than it will already reach.” For participants like Hamish, testing offered the potential for linkages between individual behavior and population-level disease dynamics. But the scale-expanding effect of such action was sometimes accompanied by uncertainty about the extent to which individual behavior might impact on higher-order calculations in general. As Hamish went on to reflect, “Although I don’t know, I probably don’t make a huge difference in that equation because it’s a lot of people involved and a lot of transmissions.”

Movement between scales and categories – individual/population, or other collectives (such as “work”), between concrete persons and abstract wholes (Strathern 1992) – was not always smooth or self-evident, and required careful work to be brought into alignment within the act of testing. TestEd shared data on incidence with its participants via the project website – noting that the incidence levels and variants identified by TestEd, at the scale of the University community, could at times could be compared with those of the general population (Aitman et al. Preprint). But at the same time, the number of staff and students present on campus across the University was unknown, meaning that the proportion of staff who were TestEd users was also unknown, and as a research project, TestEd data could not be used to make decisions at the level of University governance.

**Figure 2.**
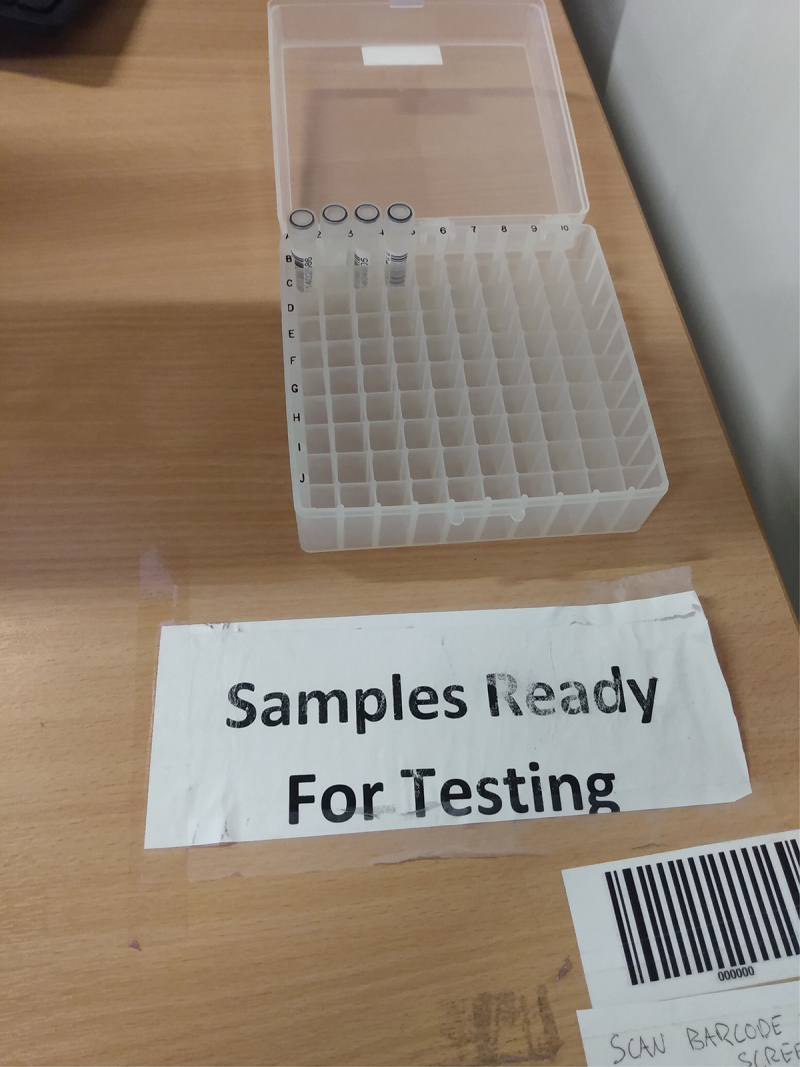
Saliva samples in a test rack for collection, Main Library (photo by Imogen Bevan, 2022).

Participants were similarly interested in the success of TestEd in picking up cases (see Bauld et al. 2023). Some referred to the visual presence of vials at testing booths as suggestive of testing levels and uptake (Figure 2). They felt concerned that their individual participation may not be having an effect at a population scale because not enough people were participating in the program. Joe, an undergraduate student in social sciences, observed:

I judge [how many people are using TestEd] by the number of vials in the tray when I go in and do it. […] You’d think with the number of students that there are at the university that there would be much more at times.

Joe’s attention to the testing practices of others when we spoke to him in December 2021 when cases were rising in Edinburgh, show his concern about the effectiveness of TestEd as a public health tool, but also a concern with the social and moral character of the testing community he had become part of. Sociologists of science have shown that the communities who participate in medical research rarely preexist the research project to which they are recruited, but are made through processes invested with moral and political values, and are better conceived as “experimental publics” (Montgomery and Pool 2017:50). At the University of Edinburgh, the existence of a testing community was felt most strongly by those who identified as part of a (both University-sited, and broader) community of scientists. Laboratory technician, Louise, described how “the natural sciences people” would happily spit in public to provide their samples, and even find this to be a “social event.”

[Y]ou’ll see someone and you’ll be like, “Oh hi,” and they’ll have their mask on, but you can see that their nose is wiggling and you know that they’re trying to build up a good bit of spit to go to the station. And then you’re like, “Oh, are you going to go to the … ?” And they’ll be like, silent wave […]. It’s like a social event.

Like Louise, participants were most likely to feel part of an experimental “community” (Montgomery and Pool 2017:30) where they felt geographically, conceptually or interpersonally connected to TestEd. “I know from my own PhD, having to go and collect samples in general can be a bit of a pain,” one staff member sympathized, while a student spoke of a “duty” to undertake regular testing as a student of infectious diseases. This Mertonian sense of communality, focused on social collaboration and the common ownership of scientific goods (Merton 1973, also see Attenborough 2020; Kelty 2012) was expressed at once in terms of helping colleagues involved in TestEd with their research, producing a sense of pandemic solidarity for those who continued to work on campus, and as contributing in a more abstract sense to science as a public good. Esau, a PhD student in biological sciences, referred to the saliva samples as “gold,” and wondered whether they would store them for future research. “I think the pandemic will come to pass, but whatever we learn from it will be essential for the next outbreak. So I think every single data point counts,” he reflected in January 2022.

While participants were uncertain as to the links between individual acts of testing and population-level effects on public health, the link between testing and a sense of collegiality and pandemic solidarity at the scale of the University was palpable. The reconfiguration of a person’s saliva into valuable “data points” that endure through time offers possibilities for forging connections between the individual and more diffuse publics, to infectious disease science, management and preparedness in the future.

## Testing as relational technology

TestEd enabled participants to enact various desires to contribute to the public good – to pandemic management, science, to colleagues’ careers, and to a sense of community – whilst going to work, to study and to participate in the economy, which represented an ever-varying risk in terms of disease transmission. At the interpersonal level, TestEd also became a very practical tool for enacting social responsibilities and obligations toward specific others – by preventing them from passing on the virus, and its medical, social and economic ramifications. Elderly parents, and vulnerable friends or relatives figured especially strongly in people’s accounts of their motivations to test, and for a few participants, fears of long COVID extended these responsibilities to otherwise healthy friends and family without known vulnerabilities. Rory, an undergraduate student in physical sciences, told us in June 2021:

Mainly it’s my parents. I have a friend who is in one of the vulnerable groups, so mainly for other people. But in general as well, just seeing elderly people about, in the supermarket and things.

In some cases, these “vulnerable” others were strangers with whom they shared a relation of physical proximity. For Hamish (introduced above):

You don’t know who does and who doesn’t [have asthma] and I go to the cafeteria, I live in [student halls], so if I’m there and I happen to be positive and I happen to spread it to someone I don’t know but is immunocompromised or does have asthma, then that wouldn’t be particularly responsible.

As with voluntary mask wearing in the UK, testing became a way to express moral duty to others (Hanna et al. 2022), and as with the category of students as risky, the category of “vulnerable” was absorbed and domesticated (Herrick 2022). In making sure he wasn’t testing positive before having contact with others with asthma, participants like Hamish engaged with TestEd primarily as a personal diagnostic service, through which they could manage their responsibility for the risks of transmission between specific individual bodies. This was in contrast to the original public health rationale of TestEd as a screening program, through which an individual test could contribute to the control of disease at the level of the population. The motivation to test was primarily experienced as an interpersonal, rather than a biopolitical, responsibility.

While some participants undertook the recommended two tests per week, many described using a “strategic” approach – offering an alternative version of routine testing that was dependent on their social routines. Interviewees reported testing in advance of meeting vulnerable family members, often with precise timing. When we asked Louise, a laboratory technician, in December 2021, how often she had used TestEd in the last weeks, she logged into her personal TestEd platform. Checking through past results, she explained that she had tested on average twice a week but had been more “spitty” in some weeks than others. Participants were more likely to link being “spitty” to particular events, such as the start of term, an invitation to dinner at a supervisor’s house, or a visit to family in another city, rather than to rises in local or national case numbers, or to the intensification or relaxation of restrictions. In Louise’s case:

[I]f I was going to go and see my father-in-law or something, I would be more likely to do it on a Thursday and a Friday so that I would see that I was negative before I went and saw him. I have used it strategically.

Testing can be read as part of “kin work” (Di Leonardo 1987), the everyday labor that people (particularly, women) put into sustaining good family relations across households, and as “relational action” (Stasch 2009:17), in that it gives meaning to particular kinship and friend relations as especially important and worthy of attention. Other participants reported testing at the start of the week, after seeing friends at the weekend, to account for different kinds of exposure. In January 2022, Esau (introduced above) observed,

There is a group that I meet on Tuesday and there’s a group that I meet on Friday at [football grounds], and then I climb over the weekends where I use public transport. […]. So there is a lot of people at risk. So you can imagine, there is no need of me not wanting to test.

For Esau, this was in addition to living with four flatmates and commuting to the laboratory for work, at a time when social mixing was allowed yet the new more transmissible Omicron variant posed important challenges to sociability. By retrospectively screening the content of past encounters for infectivity, testing established separations between relationships and spheres normally perceived as apart (work, home, leisure) – but which now risked spilling into one another. Everyday pandemic ethics (Dannesboe et al. 2023; Farah 2023; Levine and Manderson 2021) in this context involved managing the complex crossover of social circles between kinship, friends, co-living and co-working relationships – where relations of physical proximity did not necessarily map onto relations of social and emotional proximity, and constituted an ethical problem to resolve.

People’s sense of responsibility fluctuated. While participants had a positive view of vaccination – as a moral duty toward others – some found that it complicated their interpersonal responsibilities since they perceived it as making their infectiousness, identified through symptom manifestation, harder to identify. This sense of responsibility was also amplified at particular moments, the most striking of these being the event of a positive test result. Participants’ relational labor worked in multiple directions, looking forwards in reflecting on whom to protect, and how, and backwards, to identify the source and direction of a particular disease trajectory hidden within leaky encounters.

Some TestEd participants used terms like “shocked,” “angry,” or “insulted” to describe their reaction to a positive result, which did not align with their sense of themselves as behaving responsibly and the perception that they were “doing everything you can,” as Colin, a facilities worker, put it. Testing positive came with heightened duties to minimize transmission, and highlighted cohabitation with non-kin as a contentious space for ethical labor. For example, despite the continuation of government guidance to stay home after a positive result in August 2021, Nikki, an undergraduate student, calculated that it was better to leave her shared student flat. Just ahead of the start of the new University term, she walked to her parents’ house to stay in an isolated “hobbit house” in their back garden for the duration of her self-isolation. This would protect relations with her flatmates from the awkwardness of managing her infectiousness, in terms of their own social circles, and collaboratively renegotiating what it meant to be in isolation (Long 2020). Sunan, a PhD student who found himself in a similar situation in September 2021, ended up spending money he “did not have” on ordering meal deliveries to his room to avoid damaging the health of an older unvaccinated flatmate. Others emphasized fears of disrupting flatmates’ and colleagues’ livelihoods by testing positive and forcing them to self-isolate. The tensions and complicated balance between competing political rationales – economic logics versus the logic of preventing illness – was, to a certain degree, delegated to individuals through testing programs. In the accounts of TestEd participants, these tensions were felt keenly in decisions to test and to act on test results, with implications for their relationships with specific others.

Interviewees did not use TestEd in place of the government’s PCR test and contract tracing system, free for public use when symptomatic, but more as an “early warning system,” as one participant put it. Disclosure of positive test results became a vital aspect of people’s way of expressing responsibility toward others and protecting specific relationships. When Elana, a clinical service provider, tested positive in September 2021, her first actions after booking a confirmatory NHS PCR test for the family were to inform her workplace, her children’s school, and the community of mothers through a WhatsApp chat group to enable people in her social circle to take action and test as soon as possible, rather than wait for the slower national contact tracing mechanism. She felt that, thanks to her actions, her workplace had been spared, and the infection had “stopped at me.” In another kind of protection of relationships, when Colin (above), tested positive in Spring 2021, he specifically avoided disclosing a positive TestEd result to his elderly parents and mother-in-law who he had seen earlier that week, “Because they would just worry.” To protect their emotional state, he only informed them after receiving a confirmatory NHS result.

Several interviewees expressed a sense of shame associated with disclosure. Luke, a postdoctoral researcher in the laboratory, regretted having gone to work feeling “a bit rough” in November 2021, after a negative LFD and while waiting for his TestEd result to arrive. “I did a bad thing and I can feel the judgment from some people,” he told us. Nikki (above) similarly found that, “It’s like the text of shame, having to go back and tell everyone.” Disclosure emerged as a key aspect of people’s testing ethics but was also riven with ambiguities over who one should disclose to (the state, the university, the research project, flatmates, relatives, colleagues), when (straight away, or after a confirmatory test), and for what purpose (calculating prevalence, contributing to research, protecting others).

We have shown that participants primarily perceived TestEd as a means to protect relationships with specific others – engaging with TestEd as a relational technology. People’s desires to disclose test results to the University and to the state (via the NHS), and their desires for TestEd to link up to other systems, shows that they were engaged both in practices of disease management at the level of kin, friends, flatmates and colleagues, and in the work of connecting their disease status with population-level scales of pandemic management. In attending to the quality of their social relationships, people’s desires to mitigate risks through “strategic” testing overlapped to a certain extent with public health goals, enabling forms of public health governance to unfold. Yet this responsibility also fluctuated, with the potential to cause misalignments. In the following section, we examine how participants’ sense of interpersonal responsibility shaped their attempts to configure relations between TestEd and other testing services in the pandemic ecosystem, to construct an optimized ad hoc testing system.

## Configuring a personal testing system

As a research project, TestEd was not a highly controlled experiment. Indeed, by April 2021 when TestEd was rolled out across the university campus following an initial start on some sites from January, staff and students already had access to multiple kinds of tests. This included free PCR tests through the NHS if they experienced COVID-19 specific symptoms (fever, continuous cough, loss of taste or smell) or were a close contact of a positive case. In addition, free LFDs were available at university pick-up points, pharmacies, or through the government’s online ordering system, and private testing services for international travel. While in the previous section, people’s obligations to protect specific others mostly aligned with public health goals, in this section we show that when people combine and repurpose tests according to highly personalized “use cases,” this alignment proved unstable.

Although TestEd was promoted to staff and students as an asymptomatic testing program, with a different purpose to the symptomatic PCR testing provided by the NHS, participants sometimes used the services interchangeably. Rather than using it for routine screening, some participants only engaged with TestEd when a particular event or a doubt about their COVID-19 status surfaced. Eleven of our interviewees received a positive test result from their first or second sample. These interviewees had registered some time prior to this, with the intention of testing sooner, but had not found the time. For example, Grace, a master’s student, had seen the TestEd posters in the University sports facility, and remembered this later on when a flatmate tested positive. Undergoing asymptomatic testing prior to social activities or in response to personal pandemic events enabled people to continue to participate in their social relationships, and to shield those relationships from the ever-present but invisible potential for disease transmission.

Several participants reported using TestEd when they had cold-like symptoms, especially in cases when they felt their symptoms did not meet the government’s criteria for accessing a PCR test, which in September 2021 was still limited to fever, a new persistent cough, or a loss of taste or smell. Hamish, introduced above, told us:

I did make a point of taking a TestEd test after I contracted Fresher’s Flu and after I returned and on one other occasion when a friend of mine contracted a cold. But generally it’s just been […] more of a spur of the moment thing.

Like Hamish, others reported using TestEd when they suspected exposure from someone in their immediate entourage but had not been formally contact traced. Some, like Hamish, chose to use TestEd either instead of, or in addition to, the NHS service, because they viewed it as more available, involving less hassle (“it hardly takes two minutes to fill the vial up and drop it in”), and offering a faster result. Others spoke of not wanting to needlessly “waste” government-provided tests, indicating a sense of personal responsibility for public resources. Several participants interviewed in late 2021 and early 2022 felt uncertain as to whether or not they were “symptomatic” since the new Omicron variant presented with different, often milder, and more varied symptoms than those listed by the NHS. In some such instances, TestEd provided a niche use case as a PCR testing service for people who fell between the gap of experiential symptoms and official guidance on symptom criteria.

In the case of ambiguous symptoms, TestEd’s 24-hour turnaround time compared favorably to the government-provided PCR tests. However, for the purpose of routine asymptomatic screening, participants often found the 15-minute turnaround time of the government-provided LFDs more convenient, and most interviewees used TestEd alongside, as well as, or interchangeably with these. By December 2021, Morgan, a member of support staff, told us she had come to use each test for a different purpose. She preferred to use LFDs as part of her home testing regime with her partner, and TestEd for the research element, because “the problem I found with TestEd is I do it at 8 o’clock in the morning when I go in and it’s 5 o’clock the next day before I get the result,” whereas “LFDs work brilliantly because I can take them before going into campus.”

For others, however, the noninvasive nature and accuracy of TestEd’s PCR-based saliva testing for university-goers – and the unavailability of this service for anyone outside it – meant people often viewed the service as superior to LFD testing, sometimes using it to test others by proxy. In July 2021, Rosa, for example, explained how she used her TestEd status as an indicator of her partner’s, calculating that if she tested positive, then he would know “there’s a high chance that he is positive as well.” Elana (introduced above) tested on behalf of her husband and two children to protect them from the discomforts of LFD swabbing. In becoming a member of TestEd’s “experimental public” (Montgomery and Pool 2017), Elana and Rosa received access to an otherwise scarce testing resource – noninvasive PCR testing – and creatively managed their use of the program so as to share that resource, and help others construct a COVID-19 status in dialogue with their own.

Here, the interpersonal and the public start to misalign. Personalized uses of TestEd often meant that people gave saliva samples when deemed convenient or necessary for the maintenance of specific relationships – when seeing others, managing the boundaries between different groups, or simply “being out of the house” – and did not feel tightly governed or disciplined by the University with regard to regular asymptomatic TestEd use. By December 2021, Ewan, an undergraduate student, had noticed that:

I guess it’s part of the feeling at university in general, that it’s much more you can basically do what you like. You can do TestEd and lateral flows all the time, or you can do none and pretty much nobody would know.

Ewan was comparing University testing regimes to the more stringent ones he had experienced at high school – enforced by parents – the previous year. Thus, Ewan found that, “The past couple of weeks I’ve been doing it I think once, just because I haven’t been out much; I’ve been revising a lot so I’ve been kind of busy.” Similarly, Luke, a postdoctoral researcher working in the laboratory, found it hard to remain in the “habit” of routine testing, particularly after returning from holiday, and observed that by November 2021 his TestEd use had “decayed.” During the winter break in January 2022, Lee, an undergraduate student in formal sciences told us, “I actually didn’t do the last test recently because I was staying in my dorm every day basically.” Feeling busy, being on holiday, or a lack of social life, could cause “decay” in testing routines – revealing these systems as unstable and reliant on a highly contextualized sense of risk and desire to know one’s COVID-19 status at a specific point in time, in relation to particular responsibilities toward others. Desires to achieve a COVID-19 status faster – by using an asymptomatic screening service when symptomatic – can prove problematic from a public health perspective since this risks bringing infectious individuals onto campus space. When different testing elements did not link up to protect the right persons, as we saw previously with Morgan, people reconfigured their personal testing systems accordingly.

When people’s sense of interpersonal responsibilities fluctuated, and when social routines changed, the fragile alignment between interpersonal and public health scales of relation slipped. In this section, we have shown that interpersonal responsibilities during a pandemic consisted in the management of ad hoc testing systems, shaped by different testing options, spaces, temporalities, and states of health, in order to meet the changing obligations and relations of care inherent to specific relationships with kin, friends, housemates, colleagues, and strangers. As a relational technology that could be integrated with other testing technologies (PCR and LFD tests) into a personalized ad hoc testing system, and to other forms of knowledge (bodily manifestations in oneself and others, perceptions of exposure), people used TestEd for multiple use cases, including symptomatic testing, testing before visiting vulnerable individuals, and to monitor the health status of other family members. This shows how testing, like mask wearing (Lupton et al. 2021) became, temporarily at least, the norm to express care in the exceptional time of a pandemic.

## Conclusion

“I don’t want other people to think that I was responsible for passing it on because of my negligence, if you like, or my lack of care, or my lack of effort,” Alan, a facilities worker, summarized. While tensions emerged between trying to care for proximate others, and trying to safeguard them from viral transmission – where the risks associated with touch, shared breath or any act of co-presence could breach established social norms (Baffelli and Schröer 2021,; Douglas 2021) – testing offered a temporary solution to some of the ethical and relational dangers of pandemic living.

Rather than represent a new shift, the reevaluation of interpersonal relationships as sites of risk during COVID-19 bears some parallels^15^ to the ways in which people in different settings have navigated intimate relations during HIV epidemics (e.g. Biehl et al. 2001; Rhodes and Cusick 2000; Witzel et al. 2017), or engaged in “lay epidemiology” during Ebola in West Africa (Richards 2016). In these epidemics, and others, a range of public health techniques and technologies – from masks and protective clothing, to contact tracing, quarantine, testing and its associated data – became part of people’s lives, and shaped how epidemic governance was done (e.g. Lynteris 2018; Lynteris and Poleykett 2018, MacPhail 2014; Mooney 2015). COVID-19 technologies likewise mediated relations between people, medical institutions and the state, and people’s relations to one another (e.g. Chowdhury and Basu 2021; Fearnley and Wu 2023; Hanna et al. 2022). Such technologies are often understood to work by producing “moral subjects” and “ethicalized individuals” who are “capable of exercising self-mastery, discipline, foresight, reason and self control” (Rose 1991:683) around the individual responsibility to manage contagion through bodily practices and discipline. Governmentality has been a key analytical focus for the anthropology of diagnostics (see Street and Kelly 2021). This has attended to the self-responsibilization that unfolds through people’s access to and management of tests, particularly those administered outside the clinic (e.g. Robinson 2020). The individualization of risk and responsibility for disease transmission often becomes a source of anxiety for people, with regular testing emerging as a way to embody and display the responsibility to monitor one’s health status (Witzel et al. 2017).

Our findings from the TestEd program build on research on testing and epidemics by illustrating that the relationships that are built into and generated by such programs go beyond relationships of governance. People engage with tests as relational technologies that mitigate social tension and become a valuable tool within everyday ethics – in a context where individuals felt responsible not merely for making others sick in the present and/or the longer term, but for affecting their livelihoods, care duties, and capacity to socialize. In this context, we argue, testing obligations and responsibilities were experienced as stemming from preexisting relationships to others at multiple scales, rather than being imposed by the state.

Beyond suggesting that testing is part of the labor of nurturing and sustaining kin and other kinds of relationships (Carsten 2000; Di Leonardo 1987; Edwards 2000), and put to the project of boundary-making (e.g. Twamley et al. 2023), marking out categories of persons (e.g. as “vulnerable”), and the edges of particular populations (e.g. “the student body”), we also point to how testing is used to produce disconnections. As Matthew’s comment in the opening vignette shows, a key threat that COVID-19 posed was the risk of relations pertaining to different spheres spilling into one another (e.g. colleagues, students, flatmates, friends, parents, parents-in-law), highlighting the fragility of supposedly natural divides between domains (Yanagisako and Collier 1987).

What has made testing technologies especially relevant for medical anthropologists is their propensity to serve both medical and public health objectives, to operate, often simultaneously, as tools of clinical management and tools of surveillance and health governance (Street and Kelly 2021). In this article we demonstrate that asymptomatic testing programs during COVID-19 depended on the ability of tests to cross other kinds of relational scales, in this case most notably that between interpersonal obligations and responsibilities to more diffuse publics. In China, projects of pandemic governance connected the individual actions of “ordinary people” (*laobaixing*) to the “collective” (*jitizhuyi)*, linking benefits for the collective to benefits for the individual (Cai and Mason 2022:7). In encouraging people to test for family and “those around you” (Public Health Scotland 2022:5), UK public health programs opened up spaces for alignments between testing for other persons, and testing for the “public,” building bridges between configurations of persons and interpersonal relations as concrete, and the “public” or “society” as abstract wholes (Strathern 1992). It is this capacity for alignment across scales that enables asymptomatic testing programs to work in liberal democracies as voluntary programs of epidemic governance.

To some extent, the location of the testing program on a university campus, where people felt part of a pandemic trial community and a wider scientific community, lent itself to alignments between the interpersonal and the public purposes of testing. TestEd attempted to connect the scales of the interpersonal and the public through the intermediate scale of the University – for example appealing to the idea of the university as a “community” – but did not always succeed in doing so. The individual test results generated through voluntary participation in TestEd could not link up to state mechanisms for measuring prevalence, or for decision-making at the level of the Scotland, the UK or City of Edinburgh populations, nor could they be used reliably for such at the University level. The form of governance that TestEd offered, as an intervention, relied on people’s fluctuating sense of risk in a landscape of rapidly shifting case numbers, restrictions, and political logics. Our findings reveal the instability and contextual nature of alignments between different scales (Engel 2020). Instead of using each test for a particular use case, as instructed by the authorities, we saw participants using all available tests (combining, or substituting one for another), as well as other kinds of knowledge (perceptions of symptoms, exposure, and possible confounding factors) in order to meet the diagnostic requirements of particular social scenarios. They favored accurate and fast testing processes that enabled relation action, over adherence to public health guidelines. Yet in other social situations, participants simply “forgot” about testing. When people felt that specific others and relationships were not at risk, or experienced changes in their social routine, the connection with the public purposes of testing – essential for the success of the program – was lost.

## Notes

1. For simplicity, this paper uses the term “COVID-19” to refer to both COVID-19 (the disease) and SARS-CoV-2 (the viral agent causing the disease).

2. Matthew told us he tested to protect friends rather than relatives, because he did not see his parents, who lived far from Edinburgh and had been shielding.

3. Different to England’s controversial “Test and Trace” programme, set up with private contractors.

4. Although there were some tensions regarding the directionality of care associated with other interventions, such as mask wearing. See for example Hanna et al. (2022); Zhang et al. (2023).

5. See McClaughlin et al. (2023) Appendix 3; UK Government (2021); Supplementary File 1.

6. See McClaughlin et al. (2023) Appendix 3; UK Government (2021); Supplementary File 1.

7.
https://www.ed.ac.uk/tested-covid

8. The interview guide can be viewed in Bauld et al. (2023) Data supplement 3.

9. See Bauld et al. (2023). Table 1 “Participant characteristics.”

10. See Mutesa et al. (2021) for a description of the pooled testing method (“hypercube” strategy) on which TestEd is based.

11.
https://www.ed.ac.uk/tested-covid

12. The UK-government funded Coronavirus Job Retention Scheme allowed employers to place employees on paid temporary leave between March 2020 and September 2021. See https://www.gov.uk/government/publications/the-coronavirus-job-retention-scheme-final-evaluation/the-coronavirus-job-retention-scheme-final-evaluation#:~:text=The%20scheme%20was%20launched%20in,to%20%C2%A32%2C500%20per%20month.

13. This bore similarities to the UK Government’s series of “Roadmap” documents with plans to ease restrictions, and to the UK Government’s subsequent tiered system.

14. See for example, the First Minister’s statement on 24 August 2021. https://www.gov.scot/publications/coronavirus-covid-19-update-first-ministers-statement-24-august-2021/

15. Although the political context, and what was at stake, was of course very different.
